# Association between the AHA life’s essential 8 and prediabetes/diabetes: a cross-sectional NHANES study

**DOI:** 10.3389/fendo.2024.1376463

**Published:** 2024-07-01

**Authors:** Wei Xu, Yuntao Feng, Guzalnur Abdullah, Ling Li, Ping Fang, Sijing Tang, Huanhuan Yang, Dehong Kong, Hemin Huang, Yang Wang, Ying Xue

**Affiliations:** ^1^ Department of Endocrinology and Metabolism, Tongji Hospital, School of Medicine, Tongji University, Shanghai, China; ^2^ Department of Cardiology, Tongji Hospital, School of Medicine, Tongji University, Shanghai, China

**Keywords:** Life’s Essential 8, prediabetes, diabetes, NHANES, cardiovascular health

## Abstract

**Background and aims:**

The American Heart Association (AHA) recently introduced the Life’s Essential 8 (LE8) to improve cardiovascular health (CVH). However, the association between LE8 and the risk of prediabetes or diabetes is not yet fully understood. Consequently, this study aims to assess the association between CVH, as evaluated by LE8, and the risk of prediabetes and diabetes.

**Methods and Results:**

This cross-sectional study encompassed 7,739 participants aged ≥20 years from the 2007-2018 National Health and Nutrition Examination Surveys (NHANES). The CVH of participants was evaluated using the LE8, combining four health behaviors and three health factors. Glucose metabolic status categories included normal glucose metabolism, prediabetes including isolated impaired fasting glucose, isolated impaired glucose tolerance, both IFG and IGT, and diabetes. The associations between CVH and prediabetes and diabetes were analyzed using logistic regression, linear regression, restricted cubic splines, and subgroup analyses. Among 7,739 participants, 1,949 had iIFG, 1,165 were diagnosed with iIGT, 799 were IFG+IGT, and 537 were diagnosed with diabetes. After multivariable adjustments, CVH scores were inversely associated with prediabetes and diabetes, with the most robust inverse association observed between IFG+IGT and CVH across all prediabetes subgroups. Of all CVH components not directly in the causal pathway, body mass index (BMI) had the most robust associations with prediabetes and diabetes. Subgroup analyses indicated that the negative correlation between CVH and prediabetes was stronger among those with university or higher education.

**Conclusion:**

CVH, as defined by LE8, showed a significant negative association with prediabetes and diabetes.

## Highlights

• Cardiovascular health and prediabetes/diabetes association using Life’s Essential 8.
• Cross-sectional study of 7,739 NHANES participants.
• Inverse association between cardiovascular health scores and prediabetes/diabetes.
• Body mass index and education level as key modifiers.

## 1 Introduction

The prevalence of diabetes mellitus has increased rapidly and dramatically worldwide in recent decades. In the United States, diabetes, characterized by elevated blood glucose levels, ranks among the top 10 leading causes of mortality (1). According to a current survey by the International Diabetes Federation (IDF), approximately 537 million adults worldwide have diabetes, and an additional 374 million adults have prediabetes (2), imposing a significant economic burden. In the United States, diabetes has reached epidemic proportions and affects over 10% of adults (3). It is well-established that a strong association exists between diabetes and cardiovascular disease (CVD). CVD stands as the primary cause of mortality in patients with diabetes, while diabetes serves as an independent risk factor for CVD (4). Clinical management of patients with diabetes centers on two overarching goals: enhancing glycemic control to mitigate diabetic complications (5), and modifying risk factors for complications, particularly those linked to CVD (6). Therefore, it is imperative to establish standardized metrics for assessing and continuously monitoring cardiovascular health (CVH) in patients with diabetes.

Diabetes is a progressive process, often preceded by a prediabetic stage, frequently under-diagnosed. There is no consensus on the potential link between prediabetes and the increased risk of CVD. While some studies have proposed a connection between prediabetes and an elevated risk of CVD (7–9), others have failed to establish a similar association (10, 11). In addition, prediabetes is primarily classified as impaired fasting glucose (IFG) and impaired glucose tolerance (IGT), and the relationship between these two types and CVD individually requires further exploration.

In 2010, the American Heart Association (AHA) introduced Life’s Simple 7 (LS7) to advocate for initiatives to reduce the risk of CVD. The LS7 evaluates seven modifiable and actionable metrics of CVH, including dietary intake, physical activity, smoking, body mass index (BMI), blood glucose, blood pressure (BP), and blood lipids (12). Over the past decade, the LS7 has been widely utilized and has made significant contributions to the advancement of CVH in the United States and globally. However, during this period, certain limitations of LS7 have come to light (13). In 2022, in response to these limitations, the AHA proposed Life’s Essential 8 (LE8), featuring key enhancements, including the addition of sleep quality indicators and refined scoring algorithms (13). A limited number of studies have found a negative correlation between LE8 and diabetes (14, 15); however, the association of LE8 with prediabetes remains uncertain.

Considering the well-established associations between diabetes and CVD, promoting CVH as a strategic approach for the prevention and management of diabetes could potentially alleviate the burden of diabetes. Previous studies have explored the relationship between LS7, LE8, and diabetes (14–18), yet no study has examined the associations between the newly launched LE8 and prediabetes. To address these research gaps, we conducted an in-depth analysis using data from the National Health and Nutrition Examination Surveys (NHANES) to comprehensively assess the connection between LE8 and various prediabetes subtypes, as well as diabetes, among US adults.

## 2 Methods

### 2.1 Study design and participants

The data were from NHANES, which offers a comprehensive health and nutrition evaluation of noninstitutionalized civilians in the United States through a stratified, multi-stage, probabilistic cluster design that ensures national representation. The National Center for Health Statistics (NCHS) in the U.S. manages NHANES, involving in-person interviews, physical examinations, and laboratory tests. The NCHS Disclosure Review Board approved the survey methodologies. The NCHS Ethics Review Board granted ethical clearance, and participants submitted written informed consent to the NCHS Ethics Review Board. Protocol details are available at https://www.cdc.gov/nchs/ahcd/ahcd_confidentiality.htm. The Ethics Committee of Shanghai Tongji Hospital provided ethical approval for this study. Data from seven NHANES cycles (1999–2018) included a total of 101,316 individuals initially. Due to missing clinical data, unknown medical history, and incomplete LE8 data, the number of participants aged 20 years and older eligible for statistical analysis in the NHANES cycles (2007-2018) was 7,739 (**Supplementary Figure 1**).

### 2.2 Assessment of CVH by LE8

The LE8 system assesses CVH by considering four health behavior scores: diet (as measured by the 2015 Healthy Diet Index from 24-hour recalls), physical activity, nicotine exposure, and sleep. It also incorporates four health factor scores: BMI, blood lipids, blood glucose, and blood pressure (13). Blood glucose was not included in the analysis as a LE8 metric, as prediabetes and diabetes were the outcomes of interest (19). Each indicator in the LE8 system is scored on a scale from 0 to 100, and the overall score is calculated as the mean of the scores. Detailed information is available in **Supplementary Table 1**. In accordance with the guidelines of the American Heart Association (AHA), LE8, including health behavior and health factors, was analyzed continuously and categorically by tertiles: high (80–100), moderate (50–79), and low (0–49) (13).

### 2.3 Assessment of prediabetes and diabetes

Prediabetes and diabetes were defined according to the 2003 American Diabetes Association (ADA) criteria (20). Diabetes was defined by one of the following criteria: (1) fasting plasma glucose (FPG) ≥126 mg/dL (7mmol/L); (2) self-reported use of hypoglycemic medications; (3) 2 h plasma glucose (2hPG) ≥200 mg/dL (11.1mmol/L); (4) self-reported diabetes. After exclusion of diabetic subjects based on the above criteria, isolated impaired glucose tolerance (iIGT) was defined as FPG <100 mg/dL (5.6mmol/L) and 2hPG 140–199 mg/dL (7.8–11mmol/L); isolated impaired fasting glucose (iIFG) was defined as FPG 100–125 mg/dL (5.6–6.9mmol/L) and 2hPG <140 mg/dL(7.8mmol/L); both IFG and IGT (IFG+IGT) were FPG 100–125 mg/dL (5.6–11mmol/L)and 2hPG 140–199 mg/dL (7.8–11mmol/L); normal glucose metabolism (NGM) referred to FBG <100 mg/dL (5.6mmol/L) and 2hPG <140 mg/dL (7.8mmol/L).

### 2.4 Covariates

Covariates included age, sex (male and female), race and ethnicity (non-Hispanic black, non-Hispanic white, Mexican American, or other races), poverty-to-income ratio (PIR) [low (<1.3), middle (1.3–3.5), and high (≥3.5)], marital status (divorced/separated/widowed, married/living with partner and never married), and education level (college graduate or above, high school or below, and college level). Insulin resistance (IR) was assessed using the homeostasis model of IR (HOMA-IR)=(fasting plasma insulin [μU/mL])×(fasting plasma glucose [mmol/L])÷22.5 (21).

### 2.5 Statistical analysis

NHANES employed design weighting to ensure the representativeness of the data. We performed weighted data analyses using relevant survey weights (MEC2yr) to generate nationally representative estimates. After applying the NHANES survey weights, our analyses provided estimates that represented approximately 59,055,054 individuals in the U.S. population. Categorical variables in the baseline information were expressed as weighted percentages, while continuous variables were presented as weighted means along with their respective confidence intervals (CIs). Group disparities were assessed by weighted variance tests and weighted chi-square tests. Additionally, we computed age-standardized prevalence estimates and 95% CIs for various score levels.

We utilized weighted multivariate logistic regression to examine the relationship between CVH with both diabetes and prediabetes. This analysis was stepwise adjusted for age, sex, marital status, education level, and race/ethnicity. We also employed restricted cubic spline regression to explore potential non-linear associations between the LE8 score and its sub-scale scores with diabetes and prediabetes. Furthermore, we conducted stratified analyses by age, sex, marital status, education level, and race/ethnicity to assess whether the association between LE8 and diabetes and prediabetes was influenced by these factors. The significance of the interaction was assessed by the interaction P-value between the LE8 score and the stratified factors.

All statistical analyses were performed using R software (Version 4.2.1, The R Foundation; http://www.R-project.org) and EmpowerStats software (Version 5.0, X&Y Solutions, Inc., Boston, MA; http://www.empowerstats.com). A significance level of *p* < 0.05 was considered statistically significant.

## 3 Result

### 3.1 General characteristics of the study population

**Tables 1** and **2** present the demographic characteristics and metabolic risk factors of the study population, categorized according to ADA criteria. The weighted average age of participants was 47.23 years, with 48.76% male, and a majority being non-Hispanic white (73.45%). Among the 7,739 participants, 1,949 had iIFG, 1,165 had iIGT, 799 had IFG+IGT, and 537 were diagnosed with diabetes. The mean CVH score for the entire study population was 66.94 ± 0.33. Stratified by glycemic metabolism status, the mean CVH scores for NGM, iIFG, iIGT, IFG+IGT, and diabetic subjects were 70.21 ± 0.39, 64.44 ± 0.53, 63.94 ± 0.85, 60.69 ± 0.67 and 59.28 ± 0.66, respectively.

**Table 1:** Weighted baseline characteristics of participants with NGM, prediabetes, and diabetes.

	Total	NGM	Prediabetes	Diabetes	P value†
No. of participants	7739	4010	3192	537	
Age, y, mean (SE)	47.23 (0.33)	42.66 (0.39)	51.58 (0.41)	61.29 (0.79)	< 0.0001
Age, n (%)					< 0.0001
20–44	3443 (45.21)	2328 (56.92)	1049 (33.36)	66 (14.25)	
45–64	2756 (39.13)	1229 (34.00)	1323 (45.72)	204 (42.22)	
≥ 65	1540 (15.66)	453 (9.07)	820 (20.91)	267 (43.54)	
Sex, n (%)					< 0.0001
Male	3802 (48.76)	1704 (42.50)	1815 (57.03)	283 (50.97)	
Female	3937 (51.24)	2306 (57.50)	1377 (42.97)	254 (49.03)	
Race, n (%)					< 0.0001
Non-Hispanic White	3863 (73.45)	1959 (72.35)	1615 (74.36)	289 (77.87)	
Non-Hispanic Black	1366 (8.64)	799 (9.90)	477 (6.98)	90 (8.17)	
Mexican American	1131 (7.35)	526 (6.85)	522 (8.03)	83 (7.41)	
Otder Race	1379 (10.55)	726 (10.89)	578 (10.62)	75 (6.55)	
Education level, n (%)					< 0.0001
High school or less	3333 (35.80)	1503 (31.48)	1528 (39.96)	302 (48.68)	
Some college	2340 (31.46)	1292 (32.43)	911 (30.65)	137 (27.62)	
College graduate or above	2066 (32.75)	1215 (36.09)	753 (29.39)	98 (23.70)	
Marital status, n (%)					< 0.0001
Divorced/ Separated/Widowed	1533 (16.62)	682 (14.51)	679 (17.75)	172 (29.76)	
Married/Living witd a partner	4790 (66.40)	2362 (64.07)	2113 (70.23)	315 (61.60)	
Never married	1416 (16.98)	966 (21.42)	400 (12.02)	50 (8.64)	
Poverty-to-income ratio, n (%)					0.11
< 1.3	2207 (18.59)	1115 (18.53)	918 (18.03)	174 (23.28)	
1.3–3.5	2906 (35.76)	1480 (35.06)	1208 (36.36)	218 (38.46)	
> 3.5	2626 (45.65)	1415 (46.41)	1066 (45.61)	145 (38.26)	
Glucose metabolism					
Fasting glucose, mg/dl	5.51 (0.01)	5.11 (0.01)	5.89 (0.01)	6.73 (0.05)	< 0.0001
2hPG, mg/dl	6.27 (0.04)	5.23 (0.03)	6.95 (0.05)	11.72 (0.16)	< 0.0001
HbA1c (%)	5.39 (0.01)	5.27 (0.01)	5.49 (0.01)	5.93 (0.03)	< 0.0001
HOMA−IR	2.91 (0.04)	2.12 (0.04)	3.65 (0.07)	5.43 (0.25)	< 0.0001
Lipid metabolism					
Waist (cm)	97.98 (0.27)	93.61 (0.37)	102.82 (0.38)	106.79 (0.76)	
Total cholesterol, mg/dL	195.57 (0.68)	192.71 (0.85)	198.73 (1.05)	201.10 (2.26)	< 0.0001
HDL cholesterol, mg/dL	55.24 (0.25)	57.33 (0.33)	52.72 (0.40)	52.75 (0.91)	< 0.0001
LDL cholesterol, mg/dL	116.99 (0.55)	114.49 (0.72)	119.99 (0.87)	120.06 (2.16)	< 0.0001
Triglycerides, mg/dL	116.66 (1.09)	104.46 (1.09)	130.02 (1.98)	141.50 (2.98)	< 0.0001
CVH scores	66.94 (0.33)	70.21 (0.39)	63.50 (0.45)	59.28 (0.66)	< 0.0001
Healtd behaviors score	66.79 (0.45)	67.89 (0.56)	65.70 (0.60)	63.69 (0.87)	< 0.001
HEI-2015 diet score	39.24 (0.64)	39.32 (0.80)	39.08 (0.90)	39.68 (1.60)	0.95
Physical activity score	72.84 (0.72)	76.16 (0.85)	70.03 (1.14)	59.99 (2.30)	< 0.0001
Nicotine exposure score	71.05 (0.78)	71.37 (1.04)	70.40 (0.91)	72.71 (1.95)	0.29
Sleep healtd score	84.02 (0.45)	84.71 (0.55)	83.29 (0.62)	82.39 (1.26)	0.05
Healtd factors score	67.15 (0.35)	73.31 (0.45)	60.56 (0.50)	53.41 (1.15)	< 0.0001
Body mass index score	63.24 (0.53)	70.02 (0.73)	55.76 (0.82)	49.80 (1.58)	< 0.0001
Blood lipids score	65.09 (0.50)	69.88 (0.59)	59.79 (0.79)	55.70 (1.75)	< 0.0001
Blood pressure score	73.11 (0.50)	80.02 (0.66)	66.14 (0.77)	54.72 (1.66)	< 0.0001

NGM, normal glucose metabolism; CVH, cardiovascular health; FPG, fasting plasma glucose; 2hPG, 2 h plasma glucose; HbA1c, hemoglobin A1C; HOMA-IR, homeostasis model assessment for insulin resistance; HEI, healthy eating index.

Data were presented as weighted percentages or means (95% confidence intervals).

†P for differences in baseline characteristics among participants with NGM, prediabetes, or diabetes.

**Table 2:** Weighted baseline characteristics of participants with NGM, iIFG, iIGT, IFG+IGT and diabetes.

	NGM	iIFG	iIGT	IFG+IGT	Diabetes	P value†
No. of participants	4010	1949	444	799	537	
Age, y, mean (SE)	42.66 (0.39)	49.52 (0.52)	51.62 (1.10)	57.19 (0.70)	61.29 (0.79)	< 0.0001
Age, n (%)						
20–44	2328 (56.92)	754 (37.75)	140 (37.28)	155 (19.29)	66 (14.25)	< 0.0001
45–64	1229 (34.00)	834 (46.69)	162 (38.26)	327 (46.98)	204 (42.22)	
≥ 65	453 (9.07)	361 (15.55)	142 (24.46)	317 (33.73)	267 (43.54)	
Sex, n (%)						< 0.0001
Male	1704 (42.50)	1228 (62.67)	180 (38.56)	407 (51.27)	283 (50.97)	
Female	2306 (57.50)	721 (37.33)	264 (61.44)	392 (48.73)	254 (49.03)	
Race, n (%)						0.36
Non-Hispanic White	1959 (72.35)	970 (74.17)	217 (71.55)	428 (76.36)	289 (77.87)	
Non-Hispanic Black	799 (9.90)	310 (7.22)	69 (8.04)	98 (5.77)	90 (8.17)	
Mexican American	526 (6.85)	307 (7.95)	73 (8.09)	142 (8.21)	83 (7.41)	
Other Race	726 (10.89)	362 (10.65)	85 (12.32)	131 (9.65)	75 (6.55)	
Education level, n (%)						0.65
High school or less	1503 (31.48)	898 (39.20)	217 (39.56)	413 (42.27)	302 (48.68)	
Some college	1292 (32.43)	569 (30.40)	128 (32.77)	214 (30.21)	137 (27.62)	
College graduate or above	1215 (36.09)	482 (30.39)	99 (27.67)	172 (27.52)	98 (23.70)	
Marital status, n (%)						0.02
Divorced/ Separated/Widowed	682 (14.51)	363 (16.10)	96 (15.78)	220 (23.32)	172 (29.76)	
Married/Living with a partner	2362 (64.07)	1316 (71.51)	300 (71.15)	497 (66.26)	315 (61.60)	
Never married	966 (21.42)	270 (12.40)	48 (13.07)	82 (10.42)	50 (8.64)	
Poverty-to-income ratio, n (%)						0.11
< 1.3	1115 (18.53)	541 (17.20)	134 (19.47)	243 (19.57)	174 (23.28)	
1.3–3.5	1480 (35.06)	707 (34.99)	180 (40.80)	321 (37.78)	218 (38.46)	
> 3.5	1415 (46.41)	701 (47.81)	130 (39.73)	235 (42.65)	145 (38.26)	
Glucose metabolism						
Fasting glucose, mg/dl	5.11 (0.01)	5.94 (0.01)	5.23 (0.02)	6.10 (0.02)	6.73 (0.05)	< 0.0001
2hPG, mg/dl	5.23 (0.03)	5.81 (0.04)	8.87 (0.05)	9.08 (0.04)	11.72 (0.16)	< 0.0001
HbA1c (%)	5.27 (0.01)	5.46 (0.01)	5.41 (0.02)	5.61 (0.02)	5.93 (0.03)	< 0.0001
HOMA−IR	2.12 (0.04)	3.55 (0.10)	2.77 (0.12)	4.38 (0.16)	5.43 (0.25)	< 0.0001
Lipid metabolism						
Waist (cm)	93.61 (0.37)	102.47 (0.46)	98.99 (0.89)	105.85 (0.74)	106.79 (0.76)	
Total cholesterol, mg/dL	192.71 (0.85)	197.88 (1.18)	203.27 (2.37)	198.66 (1.87)	201.10 (2.26)	0.07
HDL cholesterol, mg/dL	57.33 (0.33)	52.79 (0.56)	55.16 (0.89)	51.25 (0.61)	52.75 (0.91)	0.003
LDL cholesterol, mg/dL	114.49 (0.72)	120.40 (1.04)	120.53 (1.94)	118.61 (1.69)	120.06 (2.16)	0.61
Triglycerides, mg/dL	104.46 (1.09)	123.44 (2.24)	137.85 (4.44)	143.95 (3.61)	141.50 (2.98)	< 0.0001
CVH scores	70.21 (0.39)	64.44 (0.53)	63.94 (0.85)	60.69 (0.67)	59.28 (0.66)	< 0.0001
Health behaviors score	67.89 (0.56)	65.88 (0.72)	65.46 (1.26)	65.33 (0.90)	63.69 (0.87)	0.85
HEI-2015 diet score	39.32 (0.80)	37.48 (1.06)	44.19 (2.32)	40.77 (1.64)	39.68 (1.60)	0.01
Physical activity score	76.16 (0.85)	73.52 (1.34)	61.46 (2.90)	64.95 (1.82)	59.99 (2.30)	< 0.0001
Nicotine exposure score	71.37 (1.04)	68.81 (1.23)	74.84 (1.81)	72.42 (1.65)	72.71 (1.95)	0.02
Sleep health score	84.71 (0.55)	83.70 (0.71)	81.35 (1.72)	83.18 (1.06)	82.39 (1.26)	0.4
Health factors score	73.31 (0.45)	62.53 (0.64)	61.90 (1.29)	54.49 (0.91)	53.41 (1.15)	< 0.0001
Body mass index score	70.02 (0.73)	57.14 (0.99)	59.90 (2.08)	49.82 (1.59)	49.80 (1.58)	< 0.0001
Blood lipids score	69.88 (0.59)	61.25 (0.89)	58.36 (1.79)	56.54 (1.23)	55.70 (1.75)	0.001
Blood pressure score	80.02 (0.66)	69.19 (0.97)	67.45 (1.86)	57.11 (1.51)	54.72 (1.66)	< 0.0001

NGM, normal glucose metabolism; iIFG, isolated impaired fasting glucose; iIGT, isolated impaired glucose tolerance, IFG+IGT, combined IFG and IGT; CVH, cardiovascular health; FPG, fasting plasma glucose; 2hPG, 2 h plasma glucose; HbA1c, hemoglobin A1C; HOMA-IR, homeostasis model assessment for insulin resistance; HEI, healthy eating index.

Data were presented as weighted percentages or means (95% confidence intervals).

†P for differences in baseline characteristics among participants with NGM, iIFG, iIGT, IFG+IGT and diabetes.

In multiple comparisons, significant differences were observed in CVH scores among NGM, prediabetes, and diabetes groups. The diabetes group exhibited significantly lower CVH scores compared to the NGM and prediabetes groups. Upon further comparison of the prediabetes subgroups, no significant difference was found in CVH scores between iIFG and iIGT, whereas the IFG+IGT group’s CVH score was significantly lower than those of the iIFG and iIGT groups (see **Supplementary Figure 2**). On average, patients with prediabetes or diabetes were older, less educated, and had poorer lipid profiles compared to those with NGM. Patients with IFG+IGT exhibited more pronounced metabolic deficits and more unfavorable CVD risk profiles, such as higher HOMA-IR, larger waist circumference, and altered lipid metabolism indices, compared to those with iIFG and iIGT (see **Table 2**).

When CVH was further subdivided into health behaviors (diet, physical activity, nicotine exposure, and sleep) and health factors (body mass index, blood lipids, and blood pressure), significant differences in scores were observed between the NGM, iIFG, iIGT, IFG+IGT, and diabetic groups (**Table 2**). The mean health behavior scores for the NGM, iIFG, iIGT, IFG+IGT, and diabetic subjects were 67.89 ± 0.56, 65.88 ± 0.72, 65.46 ± 1.26, 65.33 ± 0.90, and 63.69 ± 0.87, respectively; mean health factor scores were 73.31 ± 0.45, 62.53 ± 0.64, 61.90 ± 1.29, 54.49 ± 0.91, and 53.41 ± 1.15, respectively. Compared to patients with prediabetes (including iIFG, iIGT, IFG+IGT) or diabetes, those with NGM had higher health behaviors and health factors scores, except for nicotine exposure and sleep health scores, which were not statistically different.

### 3.2 Associations of CVH scores with prediabetes and diabetes

The results of the logistic regression analysis of glucose metabolism status are summarized in **Tables 3** and **4**. This comprehensive evaluation revealed a robust inverse correlation between CVH scores and both prediabetes and diabetes. After adjusting for the variables of age, sex, ethnicity, marital status, PIR, and education, for every 10-point increase in CVH scores, the odds ratios (ORs) for each category were reduced as follows: iIFG (OR 0.77, 95% CI 0.72–0.82), iIGT (OR 0.76, 95% CI 0.69–0.84), IFG+IGT (OR 0.65, 95% CI 0.60–0.70), prediabetes (OR 0.74, 95% CI 0.70–0.78), and diabetes (OR 0.63, 95% CI 0.57–0.69). Notably, the inverse correlation between IFG+IGT and CVH was more pronounced than the negative association observed with iIFG or iIGT.

**Table 3:** Association of the cardiovascular health scores with prediabetes and diabetes.

	Prediabetes				Diabetes			
	Univariable model		Multivariable model		Univariable model		Multivariable model	
	OR (95% CI)	P value	OR (95% CI)	P value	OR (95% CI)	P value	OR (95% CI)	P value
CVH score								
Per 10-point increase	0.73 (0.69,0.76)	<0.0001	0.74 (0.70,0.78)	<0.0001	0.60 (0.56,0.65)	<0.0001	0.63 (0.57, 0.69)	<0.0001
Low (0–49)	1 (Reference)		1 (Reference)		1 (Reference)		1 (Reference)	
Moderate (50–79)	0.58 (0.49,0.70)	<0.0001	0.56 (0.46,0.69)	<0.0001	0.55 (0.38, 0.80)	<0.0001	0.32 (0.20, 0.51)	0.002
High (80–100)	0.25 (0.20,0.32)	<0.0001	0.27 (0.20,0.35)	<0.0001	0.14 (0.08, 0.25)	<0.0001	0.06 (0.03, 0.12)	<0.0001
P for trend	<0.0001		<0.0001		<0.0001		<0.0001	
Health behaviors score								
Per 10-point increase	0.94 (0.91,0.98)	0.002	0.93 (0.89,0.97)	<0.001	0.90 (0.85,0.95)	<0.0001	0.88 (0.83, 0.94)	<0.001
Low (0–49)	1 (Reference)		1 (Reference)		1 (Reference)		1 (Reference)	
Moderate (50–79)	0.93 (0.79,1.09)	0.37	0.91 (0.76,1.10)	0.34	0.94 (0.70,1.26)	0.67	0.97 (0.72, 1.31)	0.86
High (80–100)	0.72 (0.60,0.88)	0.001	0.66 (0.53,0.82)	<0.001	0.53 (0.38,0.73)	<0.001	0.49 (0.33, 0.72)	<0.001
P for trend	<0.001		<0.0001		<0.0001		<0.0001	
Health factors score								
Per 10-point increase	0.72 (0.70,0.75)	<0.0001	0.76 (0.73,0.79)	<0.0001	0.62 (0.58,0.66)	<0.0001	0.67 (0.62, 0.72)	<0.0001
Low (0–49)	1 (Reference)		1 (Reference)		1 (Reference)		1 (Reference)	
Moderate (50–79)	0.52 (0.43,0.64)	<0.0001	0.55 (0.45,0.68)	<0.0001	0.35 (0.26,0.46)	<0.0001	0.40 (0.30, 0.53)	<0.0001
High (80–100)	0.20 (0.16,0.24)	<0.0001	0.26 (0.20,0.32)	<0.0001	0.06 (0.04,0.09)	<0.0001	0.11 (0.07, 0.19)	<0.0001
P for trend	<0.0001		<0.0001		<0.0001		<0.0001	

OR, Odds ratio; CI, confidence interval; CVH, cardiovascular health.

Univariable model: unadjusted model.

Multivariable model: adjusted for age (as a continuous variable), sex, race/ethnicity, poverty-to-income ratio (as a continuous variable), education levels, and marital status.

**Table 4:** Association of the cardiovascular health scores with iIFG, iIGT, and IFG+IGT.

	iIFG				iIGT				IFG+IGT			
	Univariable model		Multivariable model		Univariable model		Multivariable model		Univariable model		Multivariable model	
	OR (95% CI)	P value	OR (95% CI)	P value	OR (95% CI)	P value	OR (95% CI)	P value	OR (95% CI)	P value	OR (95% CI)	P value
CVH score												
Per 10-point increase	0.76 (0.72,0.80)	<0.0001	0.77 (0.72,0.82)	<0.0001	0.75 (0.69,0.82)	<0.0001	0.76 (0.69,0.84)	<0.0001	0.64 (0.60,0.69)	<0.0001	0.65 (0.60, 0.70)	<0.0001
Low (0–49)	1 (Reference)		1 (Reference)		1 (Reference)		1 (Reference)		1 (Reference)		1 (Reference)	
Moderate (50–79)	0.63 (0.57, 0.69)	<0.0001	0.43 (0.31,0.60)	<0.0001	0.63 (0.44,0.91)	0.01	0.66 (0.46,0.97)	0.03	0.52 (0.39,0.69)	<0.0001	0.49 (0.36, 0.67)	<0.0001
High (80–100)	0.30 (0.23,0.39)	<0.0001	0.21 (0.15,0.31)	<0.0001	0.33 (0.22,0.51)	<0.0001	0.37 (0.23,0.60)	<0.0001	0.12 (0.08,0.19)	<0.0001	0.13 (0.09, 0.21)	<0.0001
P for trend	<0.0001		<0.0001		<0.0001		<0.0001		<0.0001		<0.0001	
Health behaviors score												
Per 10-point increase	0.95 (0.91,0.99)	0.01	0.93 (0.89,0.98)	0.01	0.94 (0.88,1.00)	0.07	0.92 (0.86,0.99)	0.04	0.94 (0.89,0.98)	0.01	0.90 (0.86, 0.95)	<0.001
Low (0–49)	1 (Reference)		1 (Reference)		1 (Reference)		1 (Reference)		1 (Reference)		1 (Reference)	
Moderate (50–79)	0.92 (0.77,1.09)	0.33	0.90 (0.73,1.11)	0.30	0.81 (0.57,1.14)	0.22	0.83 (0.58,1.17)	0.28	1.04 (0.80,1.37)	0.75	0.98 (0.72, 1.33)	0.88
High (80–100)	0.74 (0.59,0.93)	0.01	0.69 (0.53,0.90)	0.01	0.67 (0.46,0.97)	0.04	0.62 (0.42,0.92)	0.02	0.71 (0.53,0.95)	0.02	0.60 (0.44, 0.82)	0.002
P for trend	0.01		0.003		0.03		0.01		0.005		<0.001	
Health factors score												
Per 10-point increase	0.76 (0.73,0.80)	<0.0001	0.79 (0.75,0.83)	<0.0001	0.76 (0.72,0.81)	<0.0001	0.79 (0.74,0.85)	<0.0001	0.63 (0.60,0.66)	<0.0001	0.67 (0.63, 0.71)	<0.0001
Low (0–49)	1 (Reference)		1 (Reference)		1 (Reference)		1 (Reference)		1 (Reference)		1 (Reference)	
Moderate (50–79)	0.61 (0.49,0.75)	<0.0001	0.61 (0.48,0.77)	<0.0001	0.47 (0.35,0.62)	<0.0001	0.51 (0.38,0.68)	<0.0001	0.40 (0.30,0.53)	<0.0001	0.45 (0.34, 0.59)	<0.0001
High (80–100)	0.26 (0.20,0.34)	<0.0001	0.32 (0.24,0.43)	<0.0001	0.23 (0.16,0.34)	<0.0001	0.30 (0.19,0.47)	<0.0001	0.06 (0.04,0.09)	<0.0001	0.10 (0.07, 0.15)	<0.0001
P for trend	<0.0001		<0.0001		<0.0001		<0.0001		<0.0001		<0.0001	

OR, Odds ratio; CI, confidence interval; CVH, cardiovascular health; iIFG, isolated impaired fasting glucose; iIGT, isolated impaired glucose tolerance; IFG+IGT, combined IFG and IGT.

Univariable model: unadjusted model.

Multivariable model: adjusted for age (as a continuous variable), sex, race/ethnicity, poverty-to-income ratio (as a continuous variable), education levels, and marital status.

To further explore the relationship between CVH scores and prediabetes as well as diabetes, participants were stratified into low, medium, and high CVH groups based on CVH scores (refer to **Table 3**). Following multivariable adjustments, participants with moderate and high CVH had significantly lower odds of developing diabetes compared to those with low CVH, with ORs of 0.29 (95% CI 0.19–0.45) and 0.04 (95% CI 0.02–0.08), respectively. Participants in the moderate and high CVH groups also exhibited a significantly lower risk of iIFG, iIGT, IFG+IGT, and prediabetes compared to those in the low CVH group. Notably, the inverse association between IFG+IGT and CVH was more pronounced in the high CVH group compared to other prediabetes subgroups, with an OR of 0.13 (95% CI 0.09–0.21) per 10-point increase in CVH, second only to diabetes (OR 0.06, 95% CI 0.03, 0.12) (see **Table 4**). Logistics regression analysis of the association between CVH scores and prediabetes/diabetes showed that as the CVH scores went from low to high, the ORs for all types of glucose metabolism disorders, including iIFG, iIGT, IFG+IGT, and diabetes, then progressively decreased (*p* for trend <0.0001) (see **Tables 3** and **4**).

**Figure 1:**
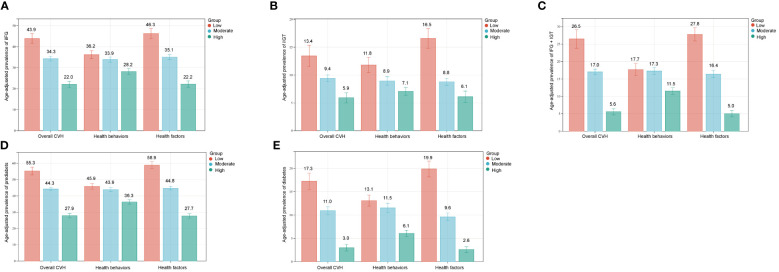
Age-adjusted prevalence of prediabetes and diabetes in different levels of CVH scores. **(A)** iIFG; **(B)** iIGT; **(C)** IFG+IGT; **(D)** prediabetes; **(E)** diabetes. Numbers at the top of the bars represent the weighted percentage. Bar whiskers represent the 95% confidence intervals. iIFG, isolated impaired fasting glucose; iIGT, isolated impaired glucose tolerance; IFG+IGT, combined IFG and IGT; CVH, cardiovascular health.

**Figure 2:**
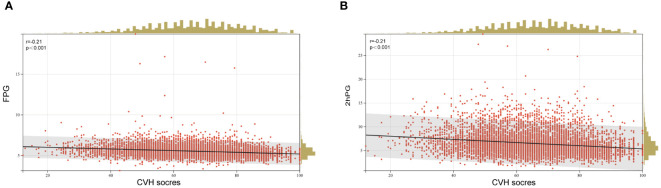
Linear regression analysis of CVH scores and **(A)** FPG and **(B)** 2hPG. FPG, fasting plasma glucose; 2hPG, 2 h plasma glucose; CVH, cardiovascular health.

Furthermore, the age-adjusted prevalence of iIFG, iIGT, IFG+IGT, prediabetes, and diabetes showed varying decreases with increasing CVH levels, health behavior scores, and health factor scores, as illustrated in **Figure 1**. When FPG and 2hPG were used as continuous glycemic measures, both FPG and 2hPG exhibited an inverse association with CVH scores as presented in **Figure 2** (β=-0.21, *p* < 0.0001 for both).

**Figure 3:**
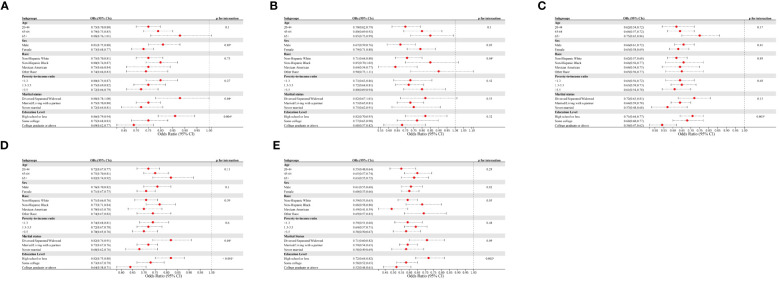
Subgroup analysis of the association of CVH scores and the presence of **(A)** iIFG; **(B)** iIGT; **(C)** IFG+IGT; **(D)** prediabetes; and **(E)** diabetes. ORs were calculated as per 10 scores increase in CVH scores. Each stratification was adjusted for age, sex, race/ethnicity, poverty-to-income ratio, education level, and marital status. iIFG, isolated impaired fasting glucose; iIGT, isolated impaired glucose tolerance; IFG+IGT, combined IFG and IGT; CVH, cardiovascular health; ORs, Odds ratios; CI: confidence interval.

We also focused on subgroups with abnormal glucose metabolism, including iIFG, iIGT, IFG+IGT, prediabetic, and diabetic subgroups (**Figure 3**). CVH scores showed a negative correlation with all of these subgroups. The negative associations between CVH scores and IFG+IGT, prediabetes, and diabetes did not significantly differ across age, gender, race, and PIR subgroups. Of note, our analysis revealed that, except for the iIGT subgroup, education level influenced the negative association between CVH scores and the risk of iIFG, IFG+IGT, prediabetes, or diabetes (*p* < 0.05 for the interaction). The inverse association between CVH scores and the risk of these four subgroups was more pronounced among participants with education levels of college graduation or above, with ORs of 0.69 (95% CI 0.62–0.77), 0.54 (95% CI 0.47–0.62), 0.64 (95% CI 0.58–0.71), and 0.52 (95% CI 0.44–0.61) for each 10-point increase in CVH scores, respectively.

### 3.3 Associations of health behaviors/health factors with prediabetes and diabetes

Logistic regression analysis revealed significant negative associations between both health factor scores and health behavior scores and the risk of prediabetes and diabetes (refer to **Tables 3** and **4**). The risk of iIFG, iIGT, IFG+IGT, prediabetes, and diabetes was significantly lower in the subgroups with moderate and high health factor scores compared with the subgroups with low health factor scores. However, the risk of iIFG, iIGT, IFG+IGT, prediabetes, and diabetes was not statistically different between the two subgroups with low health behavior scores versus moderate health behavior scores (see **Tables 3** and **4**). After adjusting for multiple confounding variables, individuals with high health behaviors scores had significantly lower odds of iIFG, iIGT, IFG+IGT, prediabetes, and diabetes, with ORs of 0.69 (95% CI 0.53–0.90), 0.62 (95% CI 0.42–0.92), 0.60 (95% CI 0.44–0.82), 0.66 (95% CI 0.53–0.82), and 0.49 (95% CI 0.33–0.72), respectively.

**Figure 4:**
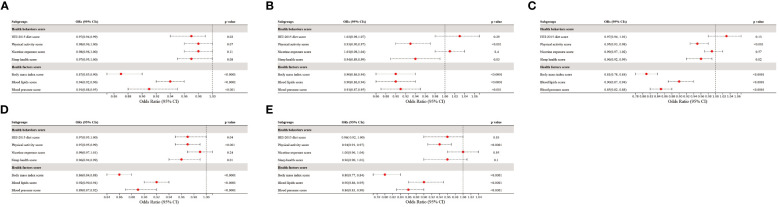
The association of CVH components with the presence of **(A)** iIFG; **(B)** iIGT; **(C)** IFG+IGT; **(D)** prediabetes; and **(E)** diabetes. ORs were adjusted for age, sex, race/ethnicity, poverty-to-income ratio, education levels, and marital status. iIFG, isolated impaired fasting glucose; iIGT, isolated impaired glucose tolerance; IFG+IGT, combined IFG and IGT; CVH, cardiovascular health; ORs, Odds ratios; CI: confidence interval.

Additionally, the associations of each CVH component with prediabetes and diabetes were shown in **Figure 4**. Notably, among the health factors, BMI score exhibited the strongest association with prediabetes and diabetes, with ORs of 0.86 (95% CI 0.84–0.88) and 0.80 (95% CI 0.77–0.84) per 10-point increase, respectively. Following BMI score, BP score, and blood lipids score were secondary factors influencing the association of CVH with prediabetes and diabetes. In contrast, health behaviors demonstrated weaker associations with prediabetes and diabetes compared to health factors. Unexpectedly, nicotine exposure score displayed no significant association with either prediabetes (p=0.24) or diabetes (p=0.95). When categorizing prediabetes into the three subtypes, the associations between CVH components and iIFG, iIGT, and IFG+IGT followed a similar pattern of correlation as in prediabetes. In conclusion, both health behaviors and health factors were negatively associated with prediabetes and diabetes, with the negative associations being more pronounced for health factors, especially body mass index.

### 3.4 Restricted cubic spline analysis

**Figure 5:**
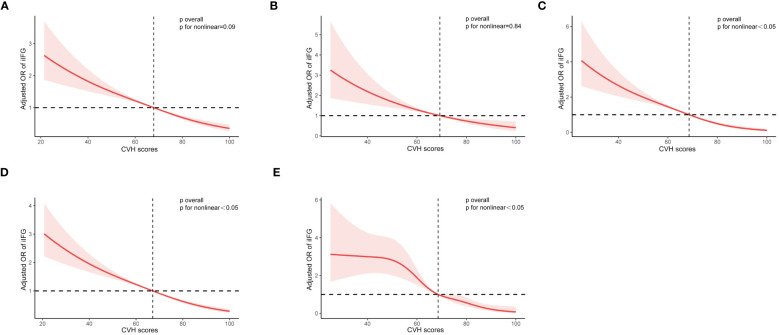
Restricted cubic curve of ORs for **(A)** iIFG; **(B)** iIGT; **(C)** IFG+IGT; **(D)** prediabetes; and **(E)** diabetes. ORs (red solid lines) and 95% confidence intervals (pink shaded areas) were adjusted for age, sex, race/ethnicity, poverty-to-income ratio, education levels, and marital status. iIFG, isolated impaired fasting glucose; iIGT, isolated impaired glucose tolerance; IFG+IGT, combined IFG and IGT; CVH, cardiovascular health; OR, Odds ratio.

As shown in **Figure 5**, restricted cubic spline (RCS) analysis with multivariate adjustment revealed that iIFG, iIGT, IFG+IGT, prediabetes, and diabetes were correlated with CVH scores (all *p* < 0.05). Among them, IFG+IGT, prediabetes, and diabetes had a significant non-linear dose-response relationship with CVH scores (*p* for nonlinear <0.05). In contrast, iIFG and iIGT had a linear dose-response association with CVH scores, with *p*-values of 0.09 and 0.84, respectively, for the non-linear test.

## 4 Discussion

This study is the first comprehensive, large-scale population-based analysis of the relationship between CVH, as defined by LE8, and prediabetes/diabetes. The findings indicate an inverse relationship between CVH scores, health behaviors, health factors, and risk of prediabetes and diabetes among U.S. adults. Among the non-causal CVH components, BMI exhibited the strongest association with prediabetes and diabetes. Notably, IFG+IGT demonstrated the strongest negative association with CVH among all prediabetes subtypes. Subgroup analyses showed that the negative association between CVH and prediabetes was stronger among individuals with a college or higher education level. These results remained robust after adjustments for various confounders, including age, sex, race/ethnicity, PIR, education level, and marital status.

Growing evidence suggests that LE8 is highly effective in assessing CVH for predicting cardiovascular disease events and cardiovascular-specific mortality in the general population and reducing premature deaths among diabetic patients (14, 22). We observed a notable decrease in CVH scores with the worsening of glucose metabolic status (from NGM to prediabetes and diabetes). This trend remained consistent when employing FBG and 2h-PG as continuous glucose measures. This negative correlation corroborates and validates previous findings in the literature. A prospective study by LU et al., including 193,846 participants aged over 40 years, demonstrated a J-shaped association between FBG, 2hPG, and cardiovascular disease events and mortality (23). In an international prospective cohort study of 18,990 participants from 21 countries, a 1 mmol/L increase in FPG or a 2.52 mmol/L increase in 2hPG was linked to an increased hazard ratio for cardiovascular events or death (1.17, 95% CI 1.13–1.22) (24). A meta-analysis pooling data from a large cohort revealed that among participants aged 55–64 years, each 1 mmol/L increase in FPG was linked to relative risks (RRs) of 1.18 (95% CI 1.08–1.29) for ischemic heart disease and 1.14 (95% CI 1.01–1.29) for stroke (25).

The LE8, as a novel assessment of CVH, remedies the shortcomings of the previous LS7 (13). The LS7, defining the original CVH component, may not adequately reflect current health behaviors and practices, particularly regarding dietary underassessment. Furthermore, the initial categorization of ideal, moderate, and poor CVH was not sufficiently sensitive to individual variations. Recent evidence has highlighted the significance of sleep in both assessing and promoting CVH. Considering the link between sleep, cardiovascular disease, and diabetes, the LS7 might not adequately capture health behaviors and physical characteristics due to the omission of sleep assessment (26, 27). Our findings underscore the inverse relationship between CVH and diabetes, consistent with prior studies based on LS7-defined CVH (16, 19, 28–30). For example, the Strong Heart Family Study (n = 1639) found that achieving a 2–3 or 4+ LS7 goal was associated with a reduced risk of diabetes, with ORs of 0.40 (95% CI 0.29–0.56) and 0.11 (95% CI 0.05–0.21), respectively (29). In the Coronary Artery Risk Development in Young Adults Study, Choi et al. observed that higher CVH scores in young adults were associated with a lower risk of diabetes and diabetic complications (30). This study was limited to black and white adults aged 20–30 years, restricting its applicability to other racial groups and to middle-aged or older populations.

Our study also revealed a significant negative correlation between CVH and prediabetes. To our knowledge, no studies have specifically addressed the correlation between CVH as assessed by LE8 and prediabetes. Prior research examining the association between LS7-based CVH and prediabetes has been limited. For instance, a study involving Japanese adults (n = 403,857) showed that an increase in non-ideal CVH metrics was associated with a higher risk of prediabetes or diabetes (31). The study utilized LS7 as an indicator for CVH assessment but omitted the impact of dietary components. Additionally, the findings of this study, based solely on Japanese participants, limit their broader applicability.

Despite the substantial evidence of the relationship between diabetes and CVH, prediabetes has garnered limited attention. Previous research has often omitted comparisons between the three distinct subtypes of prediabetes: iIFG, iIGT, and IFG+IGT. In our results, CVH scores were lower in the IFG+IGT group compared to the iIFG and iIGT groups, whereas the difference in CVH scores between the iIFG and iIGT groups was not statistically significant. With each 10-point increase in CVH scores, the odds ratios were lower in the IFG+IGT group (OR 0.65, 95% CI 0.60–0.70) compared to the iIFG group (OR 0.77, 95% CI 0.72–0.82) and iIGT group (OR 0.76, 95% CI 0.69–0.84). This association is likely related to IR. Our results revealed significantly higher HOMA-IR in the iIFG group(3.55 ± 0.10), iIGT group (2.77 ± 0.12), and IFG+IGT group (4.38 ± 0.16) than in the NGM group (2.12 ± 0.04), aligning with previous findings (32, 33). Prediabetes is heterogeneous regarding metabolic defects, hyperglycemia patterns, and cardiovascular risk. IGT is characterized by increased peripheral IR and compensated hyperinsulinemia, whereas IFG is associated with hepatic IR and excessive endogenous glucose production (34). Patients with IFG+IGT are thought to have more severe IR due to multiorgan (i.e., muscle + liver) defects (35). A cross-sectional study indicated that patients with IFG+IGT exhibited more pronounced metabolic defects, an increased likelihood of developing diabetes, and a higher CVD risk (36), which corroborates our findings. In **Figure 1** of our study, we presented a prevalence plot illustrating the correlation between CVH and prediabetes/diabetes, offering a crucial foundation for motivating patients to enhance their CVH scores. The management of prediabetes has long played a crucial role in preventing diabetes and combating diabetes-related complications. Lifestyle modification is one of the important measures. Consequently, emphasizing patients with IFG+IGT and comprehensive CVH management in this cohort can contribute to reducing the global burden of diabetes. Attaining this objective will require concerted efforts by individuals, the healthcare sector, and society to actively improve the health of the population.

Among non-direct causal risk factors, BMI exhibited the strongest association with both prediabetes (OR 0.86, 95% CI 0.84–0.88) and diabetes (OR 0.80, 95% CI 0.77–0.84), aligning with previous findings regarding BMI’s significant impact on the risk of prediabetes and diabetes (16, 29, 31). Over the past three decades, an increase in BMI has been identified as a key contributor to the escalating prevalence of diabetes in the United States (28). Programs like the Diabetes Prevention Program (DPP) have illustrated that sustained weight loss plays a significant role in diminishing diabetes risk and enhancing cardiometabolic health (37). Adipose tissue, functioning as an active endocrine organ, secretes substantial quantities of cytokines and bioactive mediators, influencing insulin sensitivity, inflammation, coagulation, and ultimately, atherosclerosis. This phenomenon likely contributes to the strong association between BMI and the development of diabetes (38).

Notably, subgroup analyses revealed a more pronounced association between CVH scores and prediabetes/diabetes among participants with a university degree or higher education (interaction *p* < 0.01). McWilliams et al. reported that diabetic adults with lower education levels exhibited significantly poorer glycemic control (39). This disparity may stem from individuals with higher education levels having improved access to comprehensive diabetes medical care and an increased capacity to assimilate and comprehend new information related to diabetes management (40). Although the underlying mechanisms remain unclear, interventions should be tailored to the specific needs of the target community to improve CVH. Unexpectedly, the association between health behavior scores and prediabetes/diabetes was less pronounced than the association between health factor scores and prediabetes/diabetes. For example, smoking did not show a significant association with either prediabetes or diabetes. This could be attributed to individuals with prediabetes or diabetes refraining from making positive lifestyle changes, such as smoking cessation and increased physical activity, until after the diagnosis is confirmed.

### 4.1 Strengths and limitations

The main strength of our study was that we explored, for the first time, the association between CVH, as defined by LE8, and prediabetes, including its three distinct subtypes. Additionally, we conducted extensive stratified analyses based on sociodemographic characteristics and risk factors for diabetes/prediabetes. However, this study had certain limitations. Firstly, it was challenging to establish a causal relationship between LE8 and prediabetes/diabetes owing to the cross-sectional design of the study. Secondly, individuals diagnosed with prediabetes or diabetes may have implemented necessary lifestyle changes, however, due to the cross-sectional design, we were unable to explore the causal relationship between lifestyle changes and risk of prediabetes/diabetes, which can be better explored in future large follow-up cohort studies. Thirdly, the diagnosis of diabetes partially relied on self-reporting, potentially leading to misclassification and estimation bias. Fourthly, the exclusion of participants with an unknown medical history or incomplete LE8 data might have led to a reduced study population and potential selection bias.

## 5 Conclusion

In conclusion, our study demonstrated a significant negative association between CVH, as defined by the LE8 score, and prediabetes/diabetes among US adults. Elevated CVH levels were associated not only with reduced risks of diabetes but also with lower risks of prediabetes. These findings offer robust evidence supporting the potential of the LE8 score in mitigating the burden of diabetes.

## Data availability statement

The datasets presented in this study can be found in online repositories. The names of the repository/repositories and accession number(s) can be found in the article/**Supplementary Material**.

## Ethics statement

The studies involving humans were approved by National Center for Health Statistics Ethics Review Board. The studies were conducted in accordance with the local legislation and institutional requirements. The participants provided their written informed consent to participate in this study.

## Author contributions

WX: Conceptualization, Software, Validation, Visualization, Writing – original draft, Writing – review & editing. YF: Writing – original draft, Writing – review & editing. GA: Data curation, Investigation, Writing – original draft, Writing – review & editing. LL: Conceptualization, Data curation, Formal analysis, Investigation, Writing – original draft. PF: Conceptualization, Data curation, Formal analysis, Funding acquisition, Investigation, Methodology, Project administration, Resources, Software, Supervision, Validation, Visualization, Writing – original draft. ST: Conceptualization, Data curation, Investigation, Methodology, Software, Writing – original draft. HY: Conceptualization, Software, Writing – original draft. DK: Data curation, Investigation, Software, Writing – original draft. HH: Investigation, Software, Writing – original draft. YW: Data curation, Methodology, Software, Writing – original draft. YX: Data curation, Funding acquisition, Resources, Supervision, Writing – original draft, Writing – review & editing.
