# Supplementary material for: Association between the AHA life’s essential 8 and prediabetes/diabetes: a cross-sectional NHANES study
Source: Front Endocrinol (Lausanne). 2024 Jul 1;15:1376463. doi: 10.3389/fendo.2024.1376463 (PMC11289523; doi:10.3389/fendo.2024.1376463)
Supplement: Supplementary file 1 [file DataSheet_1.docx]

**Additional MATERIALS**

Participants extracted from NHANES

1999-2018 (n= 101,316)

Excluded:

Participants with missing data regarding lipid metabolism (n=183)

Excluded:

Participants with missing data on medical history, marital status, household income or educational status (n = 1445);

Excluded:

Participants with age <20 years (n=42,252).

Excluded:

Participants with missing data regarding glucose metabolism (n=21,496)

Excluded:

Participants with missing values on life’s essential 8 (n=28,201)

Included participants (n= 7,922)

Eligible participants for analysis (n=7739)

Included participants (n= 59,064)

Included participants (n=57,619)

Included participants (n=28,418)

**Figure S1. Flowchart of the sample selection from NHANES 2007-2018**


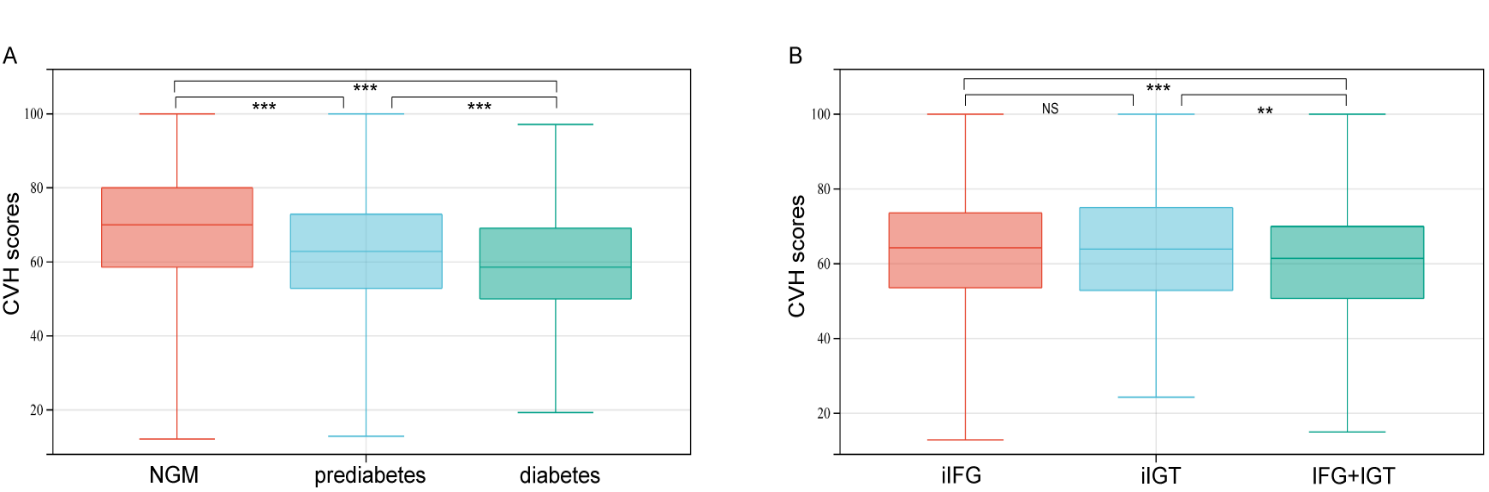


**Figure S2. CVH of NGM, prediabetes and diabetes.**

(A) NGM, prediabetes and diabetes; (B) iIFG, iIGT and IFG+IGT **p＜0.01, ***p＜0.001. NGM, normal glucose metabolism; iIFG, isolated impaired fasting glucose; iIGT, isolated impaired glucose tolerance; IFG+IGT, combined IFG and IGT; CVH, cardiovascular health.

**Table S1 Definition and scoring approach for the American Heart Association’s Life’s Essential 8 score.**

| Domain | CVH Metric | Measurement | Quantification and Scoring of CVH Metric |
| --- | --- | --- | --- |
| Health Behaviors | Diet | Healthy Eating Index-2015 diet score percentile | Quantiles of DASH-style diet adherence  **Scoring (Population):**  Points Quantile  100 ≥95^th^ percentile (top/ideal diet)  80 75^th^ – 94^th^ percentile  50 50^th^ – 74^th^ percentile  25 25^th^ – 49^th^ percentile  0 1^st^ – 24^th^ percentile (bottom/least ideal quartile) |
|  | Physical activity | Self-reported minutes of moderate or vigorous physical activity per week | **Metric:** Minutes of moderate (or greater) intensity activity per week  **Scoring:**  Points Minutes  100 ≥150  90 120 – 149  80 90 – 119  60 60 – 89  40 30 – 59  20 1 – 29  0 0 |
|  | Nicotine exposure | Self-reported use of cigarettes or inhaled nicotine-delivery system (NDS) | **Metric:** Combustible tobacco use and/or inhaled NDS use; or secondhand smoke exposure  **Scoring:**  Points Status  100 Never smoker  75 Former smoker, quit ≥5 yrs  50 Former smoker, quit 1 - <5 yrs  25 Former smoker, quit <1 year, or currently using inhaled NDS  0 Current smoker  Subtract 20 points (unless the score is 0) if there is an indoor smoker in the home |
|  | Sleep health | Self-reported average hours of sleep per night | **Metric:** Average hours of sleep per night  **Scoring:**  Points Level  100 7 – <9  90 9 – <10  70 6 – <7  40 5 – <6 or ≥10  20 4 – <5  0 <4 |
| Health Factors | Body mass index | Body weight (kg) divided by height squared (m^2^) | **Metric:** Body mass index (kg/m^2^)  **Scoring:** Points Level 100 <25  70 25.0 – 29.9  30 30.0 – 34.9  15 35.0 – 39.9  0 ≥40.0 |
|  | Blood lipids | Plasma total and HDL-cholesterol with calculation of non-HDL-cholesterol | **Metric:** Non-HDL-cholesterol (mg/dL)  **Scoring:**  Points Level  100 <130  60 130 – 159  40 160 – 189  20 190 – 219  0 ≥220  If drug-treated level, subtract 20 points |
|  | Blood glucose | Fasting blood glucose or casual hemoglobin A1c | **Metric:** Fasting blood glucose (mg/dL) or Hemoglobin A1c (%)  **Scoring:**  Points Level  100 No history of diabetes and FBG <100 (or HbA1c < 5.7)  60 No diabetes and FBG 100 – 125 (or HbA1c 5.7-6.4) (Pre-diabetes)  40 Diabetes with HbA1c <7.0  30 Diabetes with HbA1c 7.0 – 7.9  20 Diabetes with HbA1c 8.0 – 8.9  10 Diabetes with Hb A1c 9.0 – 9.9  0 Diabetes with HbA1c ≥10.0 |
|  | Blood pressure | Appropriately measured systolic and diastolic blood pressure | **Metric:** Systolic and diastolic blood pressure (mm Hg)  **Scoring:**  Points Level  100 <120 or <80 (Optimal)  75 120-129 or <80 (Elevated)  50 130-139 or 80-89 (Stage I hypertension)  25 140-159 or 90-99  0 ≥160 or ≥100  Subtract 20 points if treated level |
