# Supplementary figures and images for: Association between the AHA life’s essential 8 and prediabetes/diabetes: a cross-sectional NHANES study
Source: Front Endocrinol (Lausanne). 2024 Jul 1;15:1376463. doi: 10.3389/fendo.2024.1376463 (PMC11289523; doi:10.3389/fendo.2024.1376463)

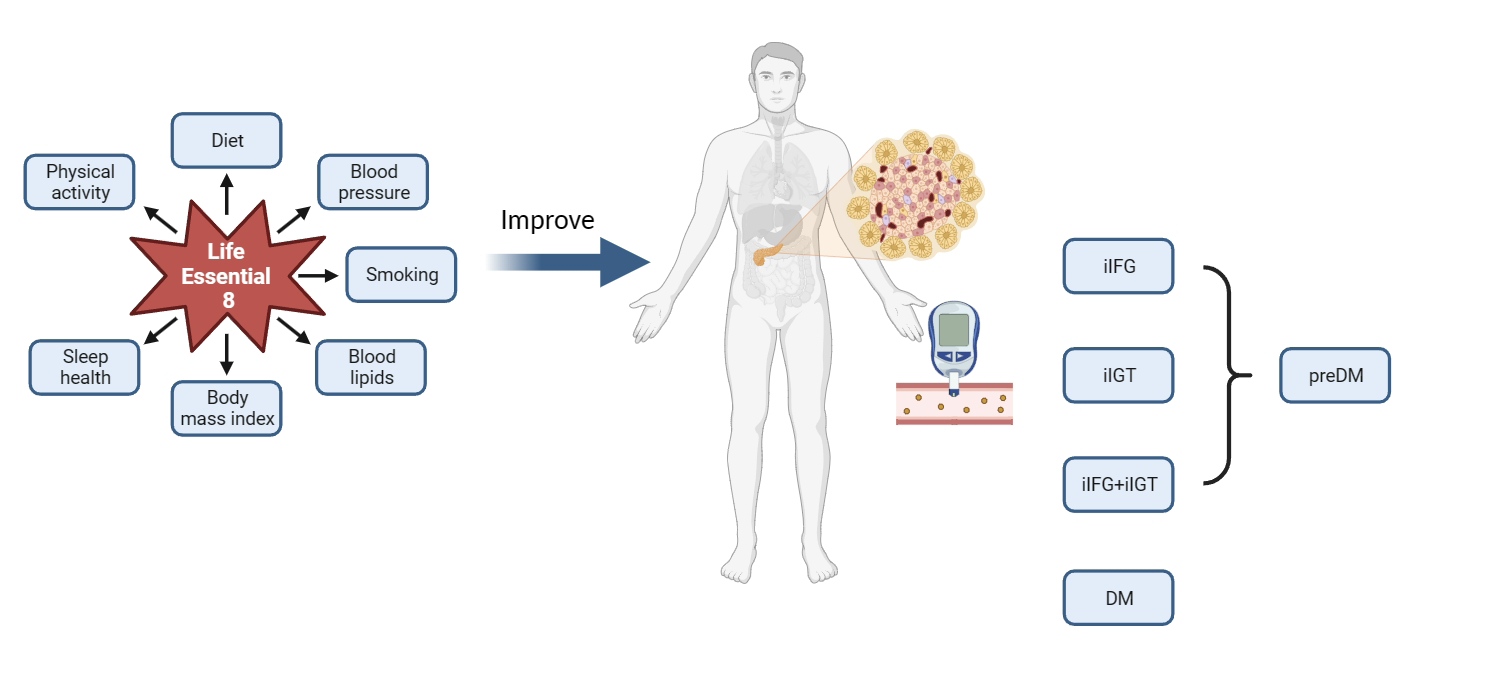

Supplement: Supplementary file 2 [file Image_1.tif]
